# Aqueous *Artemisia herba-alba* Asso Preparation as a Botanical Adjunct to Dapagliflozin: Enhanced Glycemic Control in Streptozotocin-Induced Diabetic Rats

**DOI:** 10.3390/pharmaceutics18070900

**Published:** 2026-07-22

**Authors:** Mohammad M. Hailat, Mustafa M. Al-Karkhi, Nisreen T. Al-Qaisi, Marwan Shalash, Wael Abu Dayyih, Wafa Hourani, Mohammad Abu Assab, Ahmed Bassam Farhan, Abdul Rahman Abu Dayyih, Shorouq B. Talalah, Afnan B. Talalah

**Affiliations:** 1 Faculty of Pharmacy, Al-Zaytoonah University of Jordan, Amman 11733, Jordan; m.hailat@zuj.edu.jo; 2 College of Pharmacy, Al-Kitab University, Kirkuk 36001, Iraq; mustafa.mohammed@uoalkitab.edu.iq; 3 Faculty of Pharmacy, Mutah University, Al-Karak 61710, Jordan; nisreenqaisi@hotmail.com (N.T.A.-Q.);; 4 Department of Pharmaceutics and Pharmaceutical Technology, Faculty of Pharmacy, Zarqa University, Zarqa 13111, Jordan; 5 Department of Pharmaceutical Sciences, Faculty of Pharmacy, Philadelphia University, Amman 19392, Jordan; whourani@philadelphia.edu.jo (W.H.); abdulrahman_ad@yahoo.com (A.R.A.D.); 6 Faculty of Pharmacy, Zarqa University, Zarqa 13110, Jordan; mabuassab@zu.edu.jo; 7 Faculty of Pharmacy, University of Baghdad, Baghdad 10071, Iraq

**Keywords:** natural product, phytochemical characterization, SGLT2 inhibitor, DPP-IV inhibition, combination therapy, glycated hemoglobin, type 2 diabetes mellitus, botanical adjunct, complementary medicine, Jordanian medicinal plants

## Abstract

**Background/Objectives**: optimization of the therapeutic efficacy of antidiabetic agents is still a great challenge in the management of type 2 diabetes mellitus (T2DM), and attention is becoming more and more focused on the use of adjunctive agents that help target the drug action beyond the classic glycemic target. This study examined the effect of co-administration of an *Artemisia herba-alba* Asso (Asteraceae) aqueous extract on the glucose-lowering effect of dapagliflozin, a sodium–glucose cotransporter-2 (SGLT2) inhibitor. **Methods**: qualitative phytochemical screening and total phenolic and total flavonoid content were used to characterize the aqueous preparation. Male Wistar albino rats were divided into eight groups (10 rats each): four healthy and four diabetic rats induced with streptozotocin (STZ). Vehicle control, oral administration of an aqueous *A. herba-alba* preparation (0.39 g/kg twice daily), dapagliflozin (0.143 mg/kg/day), or a combination of both was given for 30 days. At baseline and days 7, 14, 21, and 30, glycated hemoglobin (HbA1c) was measured by nephelometry. Parallel fasting blood glucose (FBG) was monitored. **Results**: the diabetic animals treated with the combination regimen showed the greatest and most sustained decrease in HbA1c (from 4.42 ± 0.40% at baseline to 3.44 ± 0.17% at day 30; *p* < 0.001) and the greatest decrease in FBG (from 186.0 to 122.2 mg/dL). *A. herba-alba* monotherapy had little effect on HbA1c and had minimal effect on FBG (214.9 to 217.6 mg/dL); dapagliflozin monotherapy had a modest effect on HbA1c. The untreated diabetic controls (*n* = 10) showed a progressive increase in HbA1c from 4.45 ± 0.60% at 72 h post-induction to 11.90 ± 0.99% by day 30 (*p* < 0.001), consistent with delayed hemoglobin glycation relative to the rise in blood glucose. The repeated-measures ANOVA showed a highly significant interaction between time and treatment (*p* < 0.001). **Conclusions**: this study showed that the phytochemically defined aqueous *A. herba-alba* preparation is a beneficial adjunct to SGLT2 inhibitor therapy, an effect not observed with either treatment alone.

## 1. Introduction

Type 2 diabetes mellitus (T2DM) is one of the most pressing public health issues of the present day. In 2021, the number of adults with diabetes was 537 million, which is expected to increase to 783 million by 2045 [1,2]. Microvascular and macrovascular complications are closely related to chronic hyperglycemia and are responsible for a high burden of the condition, both clinically and economically [3]. HbA1c is the single strongest indicator of long-term glycemic control and reflects glycemic exposure over the past 2–3 months, which correlates with the risk of complications across diverse populations [4,5].

Although there are a variety of drugs available to treat T2DM, a considerable percentage of individuals with T2DM do not achieve the desired HbA1c levels. In Jordan, there is a lack of good glycemic control among diabetic patients who suffer from comorbidities, such as heart failure, when they are treated with standard pharmacotherapy [6], and this has been confirmed in many other populations worldwide, including in the Middle East [7,8]. This therapeutic gap highlights the need for additional therapies to enhance the clinical benefits of available antidiabetic drugs and to move therapy beyond standard glycemic endpoints [9,10].

Sodium–glucose cotransporter-2 (SGLT2) inhibitors are a class of insulin-independent antidiabetic drugs that inhibit glucose reabsorption in the renal proximal tubule, thereby promoting urinary glucose excretion [11,12]. Of these, dapagliflozin has proven highly effective at reducing HbA1c and cardiovascular and renal outcomes in pivotal randomized trials, including DECLARE-TIMI 58 [13], DAPA-HF [14], and DAPA-CKD [15]. In a large population-based study in Scotland, Mahmoud et al. [16] demonstrated that by 2019, SGLT2 inhibitors had become the most widely prescribed add-on treatment in combination with metformin, further highlighting the global trend towards combination therapy. In patients with cardiorenal risk, there has been a paradigm shift in the treatment of diabetes; updated ADA/EASD consensus statements [17,18] and national guidelines [19] now recommend initiating SGLT2 inhibitors in this group.

In addition to traditional drug treatment, many cultures around the world have ancient herbal knowledge for glycemia control. The genus *Artemisia* L. (Asteraceae), commonly known as wormwood, comprises 200–400 species [20,21] and has long been used in the Middle East, Africa and Asia to treat glycemic disorders [22,23]. The hypoglycemic activity of different Artemisia species has been established in various preclinical and clinical studies, which is attributed to their ability to inhibit α-glucosidase and α-amylase, promote glucose uptake in cells via the GLUT-4 pathway, enhance insulin sensitivity, and modulate adipokine and inflammatory signaling [24,25,26,27,28,29,30]. *Artemisia herba-alba* Asso (Asteraceae) is a native species of Jordan that has been phytochemically characterized in several publications. Mohamed et al. [31] reviewed the chemistry and pharmacology of *A. herba-alba*, which includes over 30 compounds isolated to date. The Tunisian variant has been reported by Akrout et al. [32]. Two flavonoids—cirsilineol and a related hispidulin-7-O-glycoside—with hypoglycemic activity have been isolated from *A. herba-alba* by Salah and Jäger [33], and Bourgou et al. [34] reported additional phytochemical data on North African populations.

Sewidan et al. [35] have shown in vitro dipeptidyl peptidase-IV (DPP-IV) inhibitory activity in selected Jordanian plants (*A. herba-alba*), suggesting a potential complementary mechanism by which the plant might exert an additive effect alongside SGLT2-mediated glucose excretion. Previous studies have also demonstrated the effects of *Artemisia judaica* extracts as potential coadjuvants of glyburide [36] and metformin [37] and have identified compounds in extracts of Artemisia species that target metabolic complications of early diabetes in the liver using LC-Q-TOF-MS/MS coupled with network pharmacology methods [38]. *A. herba-alba* contains sesquiterpene lactones and flavones, such as santonin, hispidulin, cirsilineol, eupatilin and herbalbin.

However, no preclinical studies have examined the efficacy of an *A. herba-alba* extract formulated in an aqueous solvent, combined with a contemporary SGLT2 inhibitor, in achieving better glycemic control than either agent alone. To the best of our knowledge, this combination has not been evaluated in any other experimental system: we are not aware of any in vitro, cell-based, or ex vivo study in which *A. herba-alba* (or its isolated constituents) has been co-administered with an SGLT2 inhibitor. The available evidence concerns the two agents separately: in vitro DPP-IV and α-glucosidase inhibition by *A. herba-alba* and related species [27,33,35], and in vivo studies in which other Artemisia species were combined with glyburide [36] or metformin [37], rather than with an SGLT2 inhibitor. The present work, therefore, represents the first evaluation of this specific combination in any system. The present study aimed to close this gap by comparing the efficacy of dapagliflozin, an aqueous preparation of *A. herba-alba*, and their combination on the parameters HbA1c and fasting blood glucose (FBG) in streptozotocin (STZ)- induced diabetic Wistar rats during a 30-day oral treatment course. We also set out to provide translational pharmacology data to guide the development of botanical–drug combination therapeutics for glycemic optimization in T2DM.

## 2. Materials and Methods

### 2.1. Plant Material and Botanical Authentication

Fresh aerial parts (leaves) of *Artemisia herba-alba* Asso (Asteraceae), locally known as shih, were harvested from wild populations in Al-Tafila Governorate, in southern Jordan, in March 2023. The plant material was identified and authenticated by Prof. Dawud Al-Eisawi (Department of Biological Sciences, University of Jordan, Amman, Jordan), an expert in Jordanian flora [39]. A voucher specimen (PHARM-AHA-2023-96) was deposited at the Herbarium of the Faculty of Pharmacy, University of Jordan, for future reference. The leaves were collected and air-dried for 14–20 days in the shade at ambient temperature (~20–25 °C), after which they were finely ground using hand mills and stored in airtight containers in a cool, dry, light-free place.

### 2.2. Preparation of the Aqueous Extract

Dried plant material was prepared each day by adding approximately 2 g of dried plant material to 250 mL of fresh drinking water and steeping for 15–20 min using the traditional preparation method [40]. This infusion was left to cool, then filtered through Whatman No. 1 filter paper, and the aqueous extract was stored at 4 °C until used the same day. A new batch was prepared daily to maintain the stability of the bioactive constituents. A formal stability study of the infusion over 24 h at 4 °C was not performed; to preclude any influence of storage-related degradation, each batch was instead prepared fresh daily and used on the same day. Rats were orally gavaged twice daily (12 h apart) throughout the 30-day treatment period with 0.39 g/kg body weight of dried plant per administration; that is, 0.39 g/kg was given at each of the two daily gavages, corresponding to a total daily dose of 0.78 g/kg/day [40]. This dose was adopted directly from the rat study of Atasever et al. [40]; applying standard body-surface-area allometric scaling (rat K_m_ = 6, human K_m_ = 37), each 0.39 g/kg administration corresponds to an approximate human-equivalent dose of ~0.063 g/kg (~4.4 g of dried plant for a 70 kg adult, i.e., ~8.8 g/day in total), which lies within the range consumed as a traditional infusion. The twice-daily schedule was chosen to approximate the repeated daily intake of the traditional preparation and to provide more sustained exposure than a single bolus; formal pharmacokinetic profiling of the aqueous preparation in rats was not undertaken and is identified as a goal for future work.

### 2.3. Phytochemical Characterization

#### 2.3.1. Total Phenolic Content

The total phenolic content of the aqueous extracts of *A. herba-alba* was determined spectrophotometrically using the Folin–Ciocalteu colorimetric method [41], with gallic acid (Sigma-Aldrich, Darmstadt, Germany, ≥99% purity) as the reference compound. Briefly, 0.5 mL of the aqueous extract was mixed with 2.5 mL of 10% (*v*/*v*) Folin–Ciocalteu reagent and 2.0 mL of 7.5% (*w*/*v*) sodium carbonate solution. The mixture was allowed to stand in the dark at room temperature for 30 min, and its absorbance was measured at 765 nm against the reagent blank in a UV-Vis spectrophotometer. The results were presented as mg gallic acid equivalents per g of dry extract (GAE/g). All the measurements were repeated three times.

#### 2.3.2. Total Flavonoid Content

The total amount of flavonoids was determined using the aluminum chloride colorimetric assay [42], with quercetin (Sigma-Aldrich, Darmstadt, Germany, ≥98% purity) as the reference compound. A 0.5 mL aliquot of the aqueous extract was mixed with 1.5 mL of methanol, 0.1 mL of 10% aluminum chloride solution, 0.1 mL of 1 M potassium acetate, and 2.8 mL of distilled water. After 30 min of incubation at room temperature, absorbance was measured at 415 nm. Results were expressed as milligrams of quercetin equivalents per gram of dry extract (mg QE/g). All measurements were performed in triplicate.

#### 2.3.3. Qualitative Phytochemical Screening

Qualitative phytochemical tests were performed on the aqueous preparation for the detection of major secondary metabolite classes using standard protocols [43]: alkaloids were tested using Dragendorff’s and Mayer’s reagents (Sigma-Aldrich, Darmstadt, Germany) according to standard protocols; saponins by the frothing test; tannins by the ferric chloride test; terpenoids and sterols by the Salkowski reaction; cardiac glycosides by the Keller–Kiliani test; and anthraquinones by the Bornträger test. The presence of each compound class was scored according to the characteristic color or precipitate formed.

#### 2.3.4. Bioactive Marker Compounds Reported for *Artemisia herba-alba*

Quantitative chromatographic standardization of the aqueous preparation against individual marker compounds (e.g., HPLC-DAD/LC-MS quantification of santonin, hispidulin, cirsilineol, eupatilin, herbalbin, and chlorogenic acid) was not performed in the present study and is acknowledged as a limitation. Instead, putative bioactive markers were inferred from published phytochemical data on *A. herba-alba* [31,32,33,34,38,44,45]. Table 1 summarizes the major bioactive compounds previously reported for *A. herba-alba*, their structural classes, characteristic UV λ_max_ values, and their reported antidiabetic-relevant pharmacological activities. Throughout the discussion, these compounds have been cited as potential mediators of the effects observed on glycemia.

### 2.4. Drugs and Reagents

Dapagliflozin was purchased from a local community pharmacy, with prior contact with the drug company (AstraZeneca, Cambridge, UK), which provided the film-coated tablets. To prepare a homogeneous solution, one tablet was dissolved in 100 mL of distilled water, gently stirred, and the resulting dose (adjusted for rat metabolism [40]) was administered once daily for 30 days via oral gavage. The film-coated tablet was dispersed in its entirety; tablet excipients were not removed, and the administered dose was calculated based on the labeled dapagliflozin content. The possible influence of tablet excipients on drug absorption was not controlled and is acknowledged as a limitation. Streptozotocin (STZ; ≥98% HPLC, 75% α-anomer basis; Sigma-Aldrich/Merck, Darmstadt, Germany; batch S0130) was used to induce diabetes [46]. All other solvents and reagents, including Folin–Ciocalteu reagent, aluminum chloride, gallic acid, and quercetin, were of analytical grade and purchased from Sigma-Aldrich (Darmstadt, Germany).

### 2.5. Animals and Ethics Statement

A total of eighty (*n* = 80) male Wistar albino rats (7–8 weeks old, mean body weight 225 ± 25 g) were purchased from the animal breeding facility of the Applied Science University (Amman, Jordan) and housed in the aforementioned University animal facility under controlled conditions (temperature ~20 °C, relative humidity 50–60%, 12 h light/12 h dark photoperiod) with ad libitum access to a standard pellet diet and tap water. Animals were acclimatized for one week before the start of the study. The study was conducted over approximately 14 weeks, from February 2023 to May 2023. All experimental procedures were carried out in accordance with the institutional guidelines on animal use of Mutah University, which adopt the guidelines of the Federation of European Laboratory Animal Science Associations (FELASA) [47]. The experimental protocol was approved by the Institutional Animal Ethics Committee (IAEC) of Mutah University/ Faculty of Pharmacy (approval number: EC/2023-02/FP; date of approval: 5 February 2023). The study was designed and reported in accordance with the ARRIVE guidelines [48]. The ARRIVE checklist is available as Supplementary Material S1. Animals were humanely killed at the end of the 30-day treatment by an overdose (150 mg/kg body weight) of sodium pentobarbital according to the American Veterinary Medical Association (AVMA) Guidelines for the Euthanasia of Animals [49]. The orbital venous plexus was sampled under light diethyl ether anesthesia (~30–60 s) in a well-ventilated fume hood, in accordance with standard veterinary procedures.

### 2.6. Study Design and Group Allocation

**Figure 1:**
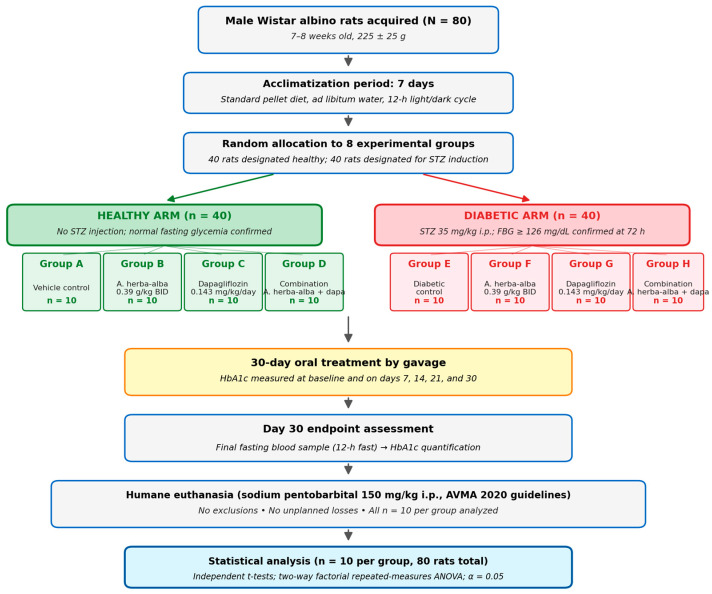
ARRIVE 2.0-compliant flow diagram of the experimental design. Eighty male Wistar albino rats were acquired, acclimatized for 7 days, and then randomly allocated to eight groups (*n* = 10 per group; total *n* = 80). Forty rats underwent streptozotocin (STZ)-induced diabetes (35 mg/kg i.p., diabetes confirmed at 72 h by FBG ≥ 126 mg/dL). All animals received the indicated treatments by oral gavage for 30 days, with HbA1c quantified at baseline and on days 7, 14, 21, and 30. No exclusions or unplanned losses occurred. Statistical analyses were performed on all *n* = 10 per group.

A total of 80 rats were divided into 8 groups (10 rats per group) (4 healthy groups (A–D) + 4 diabetic groups (E–H)). The healthy groups were: (A) vehicle control—no treatment; (B) aqueous *A. herba-alba* preparation only; (C) dapagliflozin only; and (D) combination (*A. herba-alba* + dapagliflozin). The diabetic groups were: (E) diabetic control (no treatment); (F) *A. herba-alba* only; (G) dapagliflozin only; and (H) combination (*A. herba-alba* + dapagliflozin). All groups received a standard pellet diet and tap water throughout the study. The complete animal allocation, treatment, and outcome assessment pathway is depicted in Figure 1. Throughout the study, all animals were monitored at least twice daily for signs of distress, hypoglycemia (lethargy, tremor or seizures) and morbidity. No such events, seizures or lethargy were observed in the combination groups, and no unplanned deaths were observed; no animal met any exclusion criterion, and all *n* = 10 per group were included in the analysis.

### 2.7. Diabetes Induction and Confirmation

Diabetes was induced in groups E–H by a single intraperitoneal injection of STZ (35 mg/kg body weight) dissolved in freshly prepared sodium citrate buffer (0.1 M, pH 4.5), as described previously [46]. Pancreatic β-cells take up STZ via GLUT-2 and induce selective β-cell destruction through DNA alkylation [50,51]. Diabetes was confirmed 72 h post-injection by measuring fasting blood glucose (FBG) using a commercial glucometer (JOYCOO^®^, Joycoo Health Care, Amman, Jordan; glucose oxidase biosensor assay). Animals with FBG ≥ 126 mg/dL (7.0 mmol/L) were retained in the diabetic groups [52]. The moderate STZ dose used here (35 mg/kg) is sub-maximal and is expected to cause partial rather than complete β-cell loss, leaving residual insulin secretion; this is consistent with the ability of insulin-independent glucose lowering (SGLT2 inhibition together with the phytochemical mechanisms of *A. herba-alba*) to bring fasting glucose close to the normoglycemic range in the combination group without exogenous insulin. Baseline FBG values were within the normoglycemic range across all eight groups before STZ administration (mean range: 99.67–108.7 mg/dL).

### 2.8. Blood Sampling and HbA1c Measurement

Blood samples were collected from the orbital venous plexus using plain micro-hematocrit capillary tubes after a 12 h overnight fast. Samples were collected at baseline (day 0) and on days 7, 14, 21, and 30 of treatment. Blood was placed in EDTA-anticoagulated tubes and transported to a certified medical laboratory for analysis. HbA1c was quantified using the Automatic Specific Protein Analyzer PA120 (Genrui Biotech Inc., Shenzhen, China) by the immunoturbidimetric/nephelometric method [40]. The analytical performance characteristics of this method for rat HbA1c quantification have been previously established [40].

### 2.9. Mechanistic Framework

**Figure 2:**
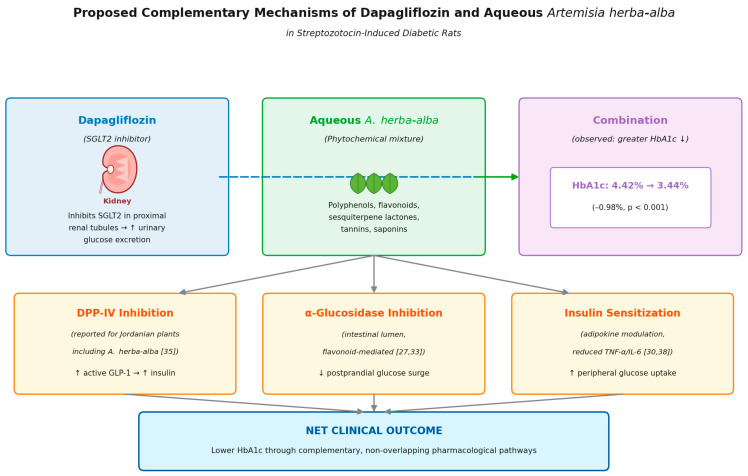
Proposed complementary mechanisms underlying the combination of dapagliflozin and an aqueous *Artemisia herba-alba* preparation in STZ-induced diabetic rats. Dapagliflozin inhibits SGLT2 in the proximal renal tubule, promoting urinary glucose excretion. *A. herba-alba* phytochemicals (polyphenols, flavonoids, sesquiterpene lactones) have been reported to act through three non-overlapping pathways relevant to glycemic control: DPP-IV inhibition (reported by [35]), intestinal α-glucosidase inhibition (flavone-mediated [27,33]), and insulin sensitization (adipokine modulation, reduced inflammatory signaling [30,38]). The net effect is hypothesized to be a greater reduction in HbA1c than achievable by either agent alone. Arrows indicate the proposed direction of action (each agent → its pathway → the net effect); the dashed blue and solid green arrows denote the contribution of dapagliflozin and *A. herba-alba*, respectively, to the combination, and ↑/↓ denote an increase or decrease.

The proposed complementary mechanisms underlying the combination of dapagliflozin with the aqueous *A. herba-alba* preparation are illustrated in Figure 2. Dapagliflozin acts at the proximal renal tubule via SGLT2 inhibition, while *A. herba-alba* phytochemicals have been reported to act through several non-overlapping pathways relevant to glycemic control: intestinal α-glucosidase inhibition by flavones [27,33], DPP-IV inhibition (reported for Jordanian *A. herba-alba* by Sewidan et al. [35]), and insulin sensitization through adipokine modulation and reduced TNF-α/IL-6 [30,38]. The net clinical outcome of these complementary, non-overlapping pathways is hypothesized to be a greater reduction in HbA1c than achievable with either agent alone.

### 2.10. Statistical Analysis

Data are presented as mean ± standard deviation (SD). Normality of distributions was assessed by the Shapiro–Wilk test and by visual inspection of histograms. Comparisons of HbA1c and FBG between healthy and corresponding diabetic groups at each time point were performed using independent-samples *t*-tests. These pre-planned pairwise *t*-tests also served as the mean-separation (post hoc) procedure when the repeated-measures ANOVA indicated a significant time × treatment interaction. A two-way factorial repeated-measures ANOVA (time × diabetic status) was used for each treatment arm, with maximum likelihood estimation via the gamma regression model and exponentiated beta.

## 3. Results

### 3.1. Phytochemical Profile of the Aqueous Artemisia herba-alba Preparation

The total phenolic and total flavonoid contents of the aqueous *A. herba-alba* preparation, determined by the methods described in Section 2.3, were 83.59 mg GAE/g and 25.70 mg QE/g of dry extract, respectively (mean ± SD, *n* = 3). Qualitative phytochemical screening revealed the presence of phenolic compounds, flavonoids, terpenoids (including sesquiterpene lactones), tannins, saponins and a cardiac glycoside, while only a small amount of alkaloids was detected. The phytochemical profile of *A. herba-alba* from Mediterranean populations reported previously [31,32,34] is consistent with that reported here: the main bioactive markers (santonin, hispidulin, cirsilineol, eupatilin, herbalbin and chlorogenic acid; Table 1) have been repeatedly identified. The complete phytochemical characterization of the preparation is summarized in Table 2.

### 3.2. Baseline Characteristics and Confirmation of Diabetes

**Figure 3:**
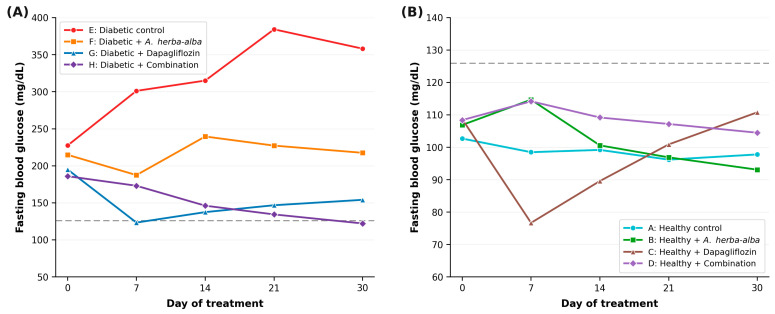
Trajectory of fasting blood glucose (FBG) levels over 30 days of treatment. (**A**) Diabetic groups (E–H): the untreated diabetic controls (E) showed progressive hyperglycemia, while the three treatment arms (F, G, H) slowed the rise to varying extents. (**B**) Healthy groups (A–D): FBG remained within the normal range throughout the study. In both panels, the gray dashed line represents the WHO diabetic threshold of 126 mg/dL. Values are group means (*n* = 10 rats per group).

There were no glycemic abnormalities before STZ administration, as baseline FBG levels were within the normal range across all eight groups (mean: 99.67–108.7 mg/dL). FBG levels in the diabetic groups rose to a post-induction range of 186.0–227.45 mg/dL within 72 h of STZ injection (approximately a doubling relative to the pre-injection normoglycemic values of 99.67–108.7 mg/dL), confirming successful induction of diabetes (Figure 3, Table 3). Independent-samples *t*-tests at baseline showed no significant differences in HbA1c between healthy and diabetic groups assigned to the no-treatment control (t = 0.282, df = 18, *p* = 0.781) or the dapagliflozin-only arm (t = 0.035, df = 18, *p* = 0.972).

### 3.3. HbA1c Changes in Diabetic Groups

**Figure 4:**
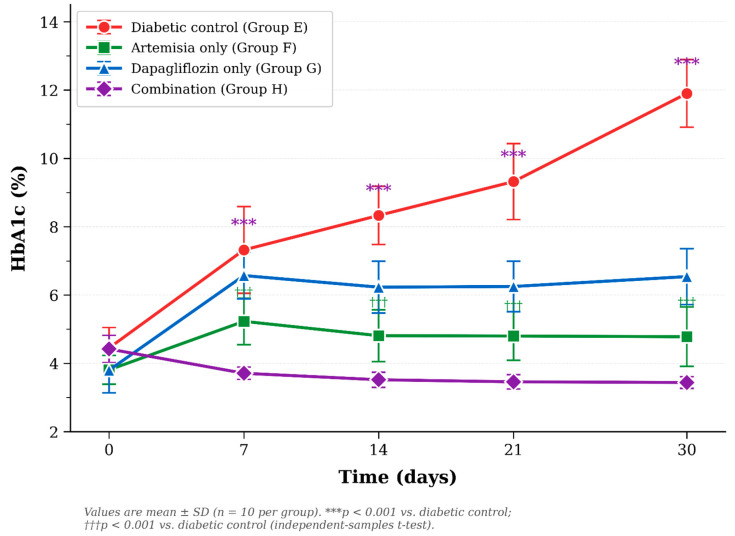
Mean glycated hemoglobin (HbA1c) values over 30 days of treatment in streptozotocin-induced diabetic rats. Group E: untreated diabetic control; Group F: aqueous *A. herba-alba* preparation (0.39 g/kg twice daily); Group G: dapagliflozin (0.143 mg/kg/day); Group H: combination therapy. Values are mean ± SD (*n* = 10 per group). *** *p* < 0.001 vs. diabetic control by independent-samples *t*-test; ††† *p* < 0.001 vs. diabetic control.

The HbA1c trajectories of the four diabetic groups are summarized in Table 4, with the corresponding trends illustrated in Figure 4 to aid visual interpretation. The untreated diabetic controls (Group E) increased steadily from 4.45 ± 0.60% at baseline to 11.90 ± 0.99% by day 30 (a +167% relative increase; *p* < 0.001), reflecting severe, unchecked hyperglycemia throughout the study.

For rats receiving the aqueous *A. herba-alba* preparation alone (Group F), baseline HbA1c was 3.81 ± 0.42%. From day 7 onwards, every HbA1c reading in this group was significantly lower than that of the diabetic controls (*p* < 0.001 at all time points). By day 30, the gap between the two groups was 7.12 percentage points in favor of the *A. herba-alba*-treated animals (4.78 ± 0.87% vs. 11.90 ± 0.99%).

In the dapagliflozin-only group (Group G), baseline HbA1c was 3.80 ± 0.66%. Compared with the diabetic controls, HbA1c was significantly lower at every post-baseline time point (*p* < 0.001 at days 7, 14, 21 and 30). At day 30, dapagliflozin-treated rats had HbA1c values 5.36 percentage points lower than the untreated diabetic controls (6.54 ± 0.82% vs. 11.90 ± 0.99%; *p* < 0.001). Dapagliflozin monotherapy nevertheless failed to prevent HbA1c from rising above its own baseline (+2.74 percentage points by day 30; Table 5), and its effect was therefore smaller than that of the combination.

For animals receiving the combination (Group H), baseline HbA1c was 4.42 ± 0.40%. At every post-baseline time point, HbA1c remained significantly lower than in untreated diabetic controls (*p* < 0.001 throughout). Over the 30 days, HbA1c actually decreased by 0.98 percentage points from baseline, reaching 3.44 ± 0.17% by day 30—a value comparable to that of healthy controls. By the final measurement, combination-treated rats were 8.46 percentage points lower than their diabetic control counterparts.

### 3.4. Comparison of Treatment Effects: Time-by-Treatment Interaction

**Figure 5:**
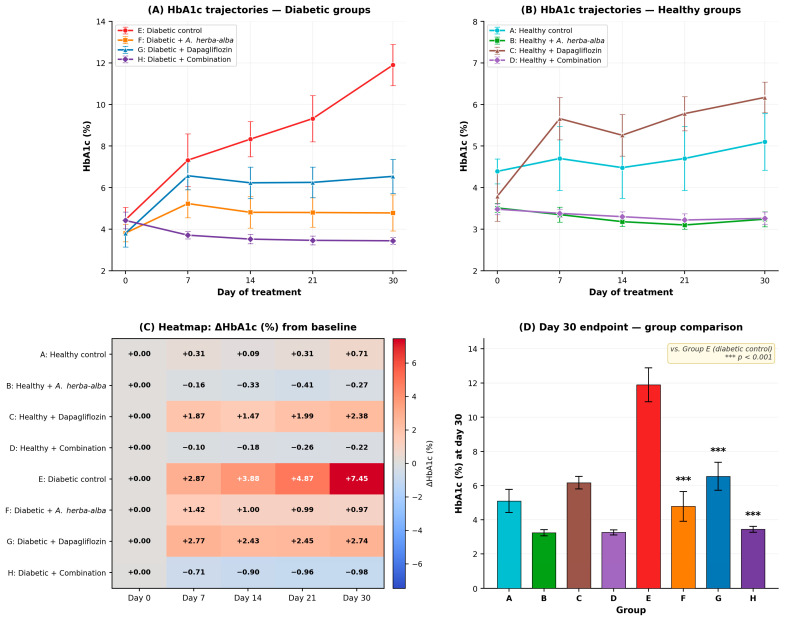
Time-by-treatment interaction on HbA1c in healthy and STZ-induced diabetic rats. (**A**) HbA1c trajectories in the four diabetic groups; (**B**) HbA1c trajectories in the four healthy groups; (**C**) heatmap of the change in HbA1c (ΔHbA1c, %) from baseline at each time point for all eight groups; (**D**) day-30 endpoint bar chart showing the final HbA1c value for each group. Values are mean ± SD (*n* = 10 per group). *** *p* < 0.001 vs. Group E (diabetic control) for Groups F, G and H. Repeated-measures ANOVA confirmed a significant time × treatment interaction (*p* < 0.001).

Repeated-measures ANOVA revealed a highly significant time-by-treatment interaction (*p* < 0.001), confirming that the four treatments shifted HbA1c in distinct directions over time. In concrete terms, the interaction means that the change in HbA1c over the 30 days depended on the treatment received: HbA1c in the untreated diabetic control rose progressively, whereas in the combination group it remained essentially flat and slightly below baseline. The *A. herba-alba* and dapagliflozin monotherapy arms followed intermediate, non-parallel trajectories. The HbA1c trajectories for diabetic and healthy groups, a heatmap of the change in HbA1c from baseline (ΔHbA1c) at each time point across all 8 groups, and the comparison at day 30 are presented in Figure 5 for a comprehensive visual summary. The heatmap (Figure 5C) clearly illustrates the progressive worsening of diabetic controls (Group E, +7.45 percentage points at day 30) versus the sustained improvement of the combination diabetic group (Group H, −0.98 percentage points at day 30). The day-30 efficacy ranking in diabetic animals was unequivocal: combination therapy (H) > *A. herba-alba* alone (F) > dapagliflozin alone (G) > diabetic control (E).

### 3.5. HbA1c in Healthy Groups

**Figure 6:**
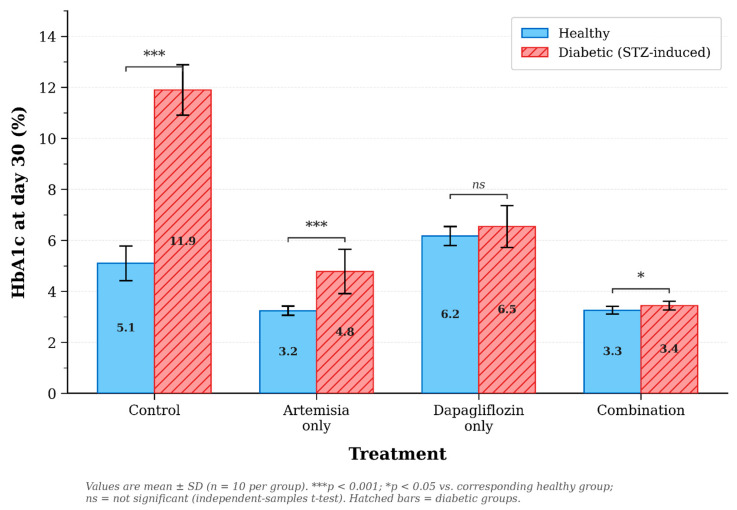
Glycated hemoglobin (HbA1c) at day 30 in healthy and diabetic rat groups receiving different treatments. Values are mean ± SD (*n* = 10 per group). *** *p* < 0.001; * *p* < 0.05; ns = not significant (independent-samples *t*-test). Hatched bars represent diabetic groups.

Healthy control rats (Group A) maintained relatively stable HbA1c levels throughout the study (4.39 ± 0.30% to 5.10 ± 0.68%). In healthy rats receiving *A. herba-alba* (Group B), HbA1c declined slightly from 3.51 ± 0.11% to 3.24 ± 0.18%. Dapagliflozin administered to healthy rats (Group C) unexpectedly increased HbA1c from 3.79 ± 0.60% to 6.17 ± 0.37%, representing a 62.8% increase relative to baseline. Combination therapy in healthy rats (Group D) maintained stable, low HbA1c values (3.48 ± 0.13% to 3.26 ± 0.15%), suggesting that the *A. herba-alba* component fully attenuated the unexpected hyperglycemic effect observed with dapagliflozin alone in normoglycemic animals (Figure 6).

### 3.6. Fasting Blood Glucose at Day 30 and Its Concordance with HbA1c

**Figure 7:**
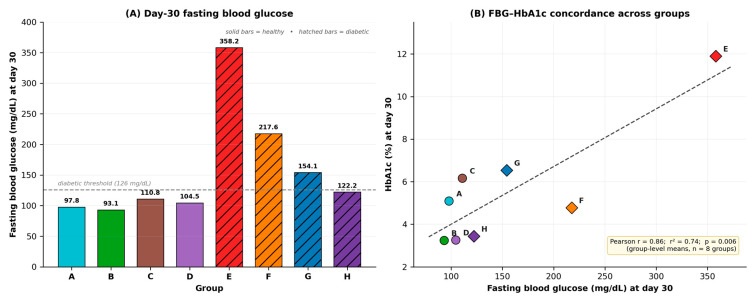
(**A**) Fasting blood glucose (FBG) on day 30 across all eight groups (group means; *n* = 10 per group); solid bars, healthy groups; hatched bars, diabetic groups. The dashed line marks the WHO diabetic threshold of 126 mg/dL. (**B**) Relationship between day-30 FBG and day-30 HbA1c across the eight group means (Pearson r = 0.86, r^2^ = 0.74, *p* = 0.006). Circles, healthy groups; diamonds, diabetic groups; the dashed line is the least-squares regression. Panel B is a descriptive group-level association (*n* = 8 group means) and not an individual-level analysis.

Because HbA1c integrates glycemia over the preceding weeks, whereas FBG reflects a single time point, the two endpoints were compared on day 30 (Figure 7). The day-30 FBG values (Figure 7A) reproduced the ranking observed for HbA1c: the untreated diabetic controls remained severely hyperglycemic (358.2 mg/dL), whereas the combination group (H) was the only diabetic arm to fall below the 126 mg/dL diabetic threshold (122.2 mg/dL). Across the eight groups, day-30 FBG and day-30 HbA1c were strongly and positively correlated (Pearson r = 0.86, r^2^ = 0.74, *p* = 0.006; Figure 7B), indicating that corresponding fasting glucose values mirrored divergent HbA1c trajectories. This correlation is a descriptive, group-level association based on eight group means and should not be interpreted as an individual-level analysis. The percentage change in FBG from baseline for every group is given in Table 6.

## 4. Discussion

The present study provides the first preclinical demonstration that an aqueous *A. herba-alba* preparation, when co-administered with dapagliflozin, produces a greater reduction in HbA1c than either treatment alone in STZ-induced diabetic rats. By combining preliminary phytochemical characterization (quantitative spectrophotometric assays for phenolic and flavonoid content, plus qualitative screening) with a well-controlled in vivo pharmacology model, this work provides meaningful data to the emerging field of antidiabetic adjunctive strategies that go beyond conventional glycemic control [6,16,18].

The combination therapy group achieved the most pronounced change in HbA1c (−0.98 percentage points from baseline; Table 5), and final values (3.44 ± 0.17%) were comparable to those of healthy controls. The glycemic benefit was observed from day 7 onwards and persisted throughout the 30-day treatment period, suggesting a rapid onset and long-term effect (Figure 5A). This was confirmed by the parallel FBG data (Figure 3A, Table 3), which showed a decrease in the combination group from 186.0 mg/dL to 122.2 mg/dL, whereas the untreated diabetic controls increased from 227.45 mg/dL to 358.2 mg/dL. Importantly, the time-by-treatment interaction was highly significant (*p* < 0.001) and indicated that the divergent trajectories were not due to chance but to true pharmacological differences between the four diabetic arms.

Hispidulin glycoside from *A. herba-alba*, which, in turn, supported the observed activity in this study. Moulahoum et al. [38] identified, using LC-Q-TOF-MS/MS and network pharmacology, active compounds from Artemisia species that target early diabetes-related metabolic complications in the liver, providing molecular-level support for the pharmacological activity observed in the present study. Importantly, Sewidan et al. [35] reported that Jordanian plants, including *A. herba-alba*, exhibit in vitro DPP-IV inhibitory activity, offering a plausible complementary mechanism by which the plant could act alongside SGLT2-mediated glucose excretion (Figure 2).

The modest effect of dapagliflozin monotherapy in this model (HbA1c 6.54 ± 0.82% at day 30, with a significant reduction relative to the diabetic control at all post-baseline time points, *p* < 0.001, that was nevertheless smaller than that produced by the combination, HbA1c remaining 2.74 percentage points above its own baseline) likely reflects the pathophysiological basis of the STZ model, which involves direct β-cell destruction rather than the insulin resistance characteristic of human T2DM [50,51,52]. SGLT2 inhibitors act independently of insulin and beta-cell function, and their effect should, in principle, remain intact in this model; however, severe and progressive hyperglycemia in untreated controls (FBG 358.2 mg/dL by day 30) may overwhelm the renal capacity for glucose excretion. The greater effectiveness of the combination treatment, in which HbA1c levels were kept at near-healthy levels, suggests that there may be complementary, and possibly more-than-additive, interactions between the botanical preparation and the SGLT2 inhibitor; because no formal synergy analysis (e.g., isobolographic or combination-index) was undertaken, we describe this as enhanced efficacy rather than synergy. So far, it has been shown that Artemisia judaica extract can improve glycemic control when added to glyburide [36] or metformin [37], supporting the use of botanicals as an adjunct to traditional antidiabetic drug therapy.

Interestingly, there was a 62.8% increase in HbA1c in healthy rats treated with dapagliflozin alone (Group C), which was completely prevented when dapagliflozin was combined with *A. herba-alba* (Group D). This finding is unexpected and runs counter to the established pharmacology of SGLT2 inhibitors. It must be interpreted with considerable caution: it relies on HbA1c alone, whereas serum insulin, glucagon, ketone bodies, urinary glucose, food intake and markers of hepatic gluconeogenesis were not measured. We therefore advance the idea of an adaptive counter-regulatory response to glucosuria, buffered by the phytochemicals of the aqueous preparation, only as a tentative hypothesis to be tested in dedicated mechanistic studies rather than as an established mechanism [38,45]. A few non-diabetic rodent studies with SGLT2 inhibitors have also shown similar paradoxical metabolic changes resulting from counteractive hepatic gluconeogenesis [11,12], which may be inhibited by the polyphenolic anti-gluconeogenic activity of *A. herba-alba* compounds [44].

From a translational perspective, these preclinical findings are hypothesis-generating rather than directly applicable to patients, particularly because the streptozotocin model reproduces β-cell-deficient hyperglycemia rather than the insulin-resistant pathophysiology that characterizes human T2DM. In a real-world clinical practice setting, a significant fraction of Jordanian patients who have diabetes, especially those with cardiovascular comorbidities (CVC), have been shown not to achieve the target HbA1c with regular pharmacotherapy [6]. Similarly, Mahmoud et al. [16] have demonstrated that the world’s pharmaceutical landscape is evolving towards combination treatments, with SGLT2 inhibitors playing a larger role in treatment. Botanical adjuncts that complement these mechanisms could offer a low-cost, clinically acceptable means (pending formal safety evaluation) further to reduce HbA1c in patients not yet at target. Beyond glycemic metrics, chronic poor glycemic control is associated with severe hypoglycemic episodes and a range of systemic and neurological sequelae, including neurodegenerative complications [53] and metabolic disturbances affecting growth factors such as insulin-like growth factor 1 (IGF-1) [54]. Approaches that achieve tighter glycemic control with a favorable safety profile could therefore offer benefits beyond glucose lowering alone.

Several limitations should be acknowledged. Each is framed here together with the specific future study needed to address it. First, although the aqueous *A. herba-alba* preparation was characterized by total phenolic and flavonoid quantification and by qualitative phytochemical screening, full quantitative chromatographic standardization against authentic reference standards (HPLC-DAD or LC-MS/MS quantification of each marker compound) was not performed in the present study. This limits batch-to-batch reproducibility.

## 5. Conclusions

In STZ-induced diabetic rats, an aqueous preparation of *A. herba-alba* Asso, administered with dapagliflozin, produced a greater reduction in HbA1c than either treatment alone, and the combination group’s levels were close to those of the healthy control group. *A. herba-alba* also showed a significant glycemic effect when used alone, and the glycemic effect of dapagliflozin alone was less in this β-cell-deficient model. The mechanisms of action of this combination effect—namely, inhibition of SGLT2 by dapagliflozin and of DPP-IV/α-glucosidase, plus insulin sensitization by phytochemicals of *A. herba-alba*—are clearly complementary, consistent with the enhanced efficacy observed for the combination rather than with formally demonstrated synergy. The results of these preclinical studies further underscore the potential of herbal preparations containing phytochemicals to be evaluated as adjuncts to SGLT2 inhibitors and as a means of extending beyond glycemic control in antidiabetic treatment. These findings warrant further investigations, such as complete chromatographic standardization, mechanistic studies, safety studies, and ultimately, clinical studies to translate these findings into evidence-based combinatorial therapy for T2DM patients.

## Figures and Tables

**Table 1:** Previously reported major bioactive compounds of *Artemisia herba-alba* Asso and their published antidiabetic activities. The literature values of UV λmax were used.

Compound	Structural Class	UV λmax (nm)	Reported Activity Relevant to T2DM	Refs.
Santonin	Sesquiterpene lactone (eudesmane)	236	Anti-inflammatory; α-glucosidase inhibition	[31,32]
Hispidulin	Flavone	275, 335	Insulin-sensitizing; PPAR-γ activation; antioxidant	[33,38]
Cirsilineol	Methylated flavone	270, 340	Hypoglycemic; α-glucosidase inhibition	[33,45]
Eupatilin	Methylated flavone	275, 340	Anti-inflammatory; improves hepatic insulin sensitivity	[38,44,45]
Herbalbin	Sesquiterpene lactone (eudesmanolide)	210–220	Reported in *A. herba-alba*; bioactivity under study	[31]
Chlorogenic acid	Hydroxycinnamic acid	325	Inhibits glucose-6-phosphatase; improves insulin sensitivity	[38,44]

**Table 2:** Phytochemical characterization of the aqueous *A. herba-alba* preparation: quantitative determination of total phenolic and total flavonoid content (mean of three determinations) and qualitative screening of the major secondary metabolite classes. +, detected; −, not detected.

Constituent/Metabolite Class	Test or Method	Result
Total phenolic content	Folin–Ciocalteu assay (quantitative)	83.59 mg GAE/g dry extract
Total flavonoid content	Aluminum chloride assay (quantitative)	25.70 mg QE/g dry extract
Alkaloids	Dragendorff’s and Mayer’s reagents	+(trace only)
Saponins	Frothing test	+
Tannins	Ferric chloride test	+
Terpenoids and sterols (incl. sesquiterpene lactones)	Salkowski reaction	+
Cardiac glycosides	Keller–Kiliani test	+
Anthraquinones	Bornträger test	−

**Table 3:** Measurement of mean fasting blood glucose (FBG) levels across all 8 groups at baseline (day 0) and after 7, 14, 21 and 30 days of treatment. Values shown are the mean (*n* = 10 rats per group). Baseline values for the diabetic groups (E to H) are post-STZ values obtained 72 h after streptozotocin injection, confirming effective induction of diabetes (all values are ≥126 mg/dL).

Group	Day 0	Day 7	Day 14	Day 21	Day 30
A: Healthy control	102.7	98.5	99.2	96.2	97.8
B: Healthy + *A. herba-alba*	106.9	114.7	100.6	96.9	93.1
C: Healthy + Dapagliflozin	108.7	76.7	89.6	100.9	110.8
D: Healthy + Combination	108.4	114.2	109.2	107.2	104.5
E: Diabetic control	227.45 ^a^	301.2	315.1	384.3	358.2
F: Diabetic + *A. herba-alba*	214.9 ^a^	187.5	239.6	227.3	217.6
G: Diabetic + Dapagliflozin	195.1 ^a^	123.4	137.5	146.8	154.1
H: Diabetic + Combination	186.0 ^a^	173.1	146.2	134.4	122.2

^a^ Post-STZ baseline, recorded 72 h after streptozotocin injection; the corresponding day-0 values for the healthy groups (A–D) are true pre-treatment baselines.

**Table 4:** Mean HbA1c values (% ± SD) across all eight groups at baseline and on days 7, 14, 21, and 30 of treatment (*n* = 10 per group).

Group	Day 0	Day 7	Day 14	Day 21	Day 30
A: Healthy control	4.39 ± 0.30	4.70 ± 0.77	4.48 ± 0.74	4.70 ± 0.77	5.10 ± 0.68
B: Healthy + *A. herba-alba*	3.51 ± 0.11	3.35 ± 0.18	3.18 ± 0.11	3.10 ± 0.10	3.24 ± 0.18
C: Healthy + Dapagliflozin	3.79 ± 0.60	5.66 ± 0.51	5.26 ± 0.50	5.78 ± 0.41	6.17 ± 0.37
D: Healthy + Combination	3.48 ± 0.13	3.38 ± 0.10	3.30 ± 0.12	3.22 ± 0.15	3.26 ± 0.15
E: Diabetic control	4.45 ± 0.60	7.32 ± 1.27	8.33 ± 0.85	9.32 ± 1.11	11.90 ± 0.99
F: Diabetic + *A. herba-alba*	3.81 ± 0.42	5.23 ± 0.68	4.81 ± 0.76	4.80 ± 0.71	4.78 ± 0.87
G: Diabetic + Dapagliflozin	3.80 ± 0.66	6.57 ± 0.68	6.23 ± 0.76	6.25 ± 0.74	6.54 ± 0.82
H: Diabetic + Combination	4.42 ± 0.40	3.71 ± 0.18	3.52 ± 0.22	3.46 ± 0.21	3.44 ± 0.17

**Table 5:** Percent change and absolute change (Δ, %) in HbA1c from baseline (day 0) at each time point for each group. Negative values indicate a decrease from baseline. The combination diabetic group (H) was the only treatment arm to produce a sustained decrease in HbA1c relative to its baseline.

Group	Day 7	Day 14	Day 21	Day 30	Δ HbA1c (Day 30)
A: Healthy control	+7.1%	+2.1%	+7.1%	+16.2%	+0.71
B: Healthy + *A. herba-alba*	−4.6%	−9.4%	−11.7%	−7.7%	−0.27
C: Healthy + Dapagliflozin	+49.3%	+38.8%	+52.5%	+62.8%	+2.38
D: Healthy + Combination	−2.9%	−5.2%	−7.5%	−6.3%	−0.22
E: Diabetic control	+64.5%	+87.2%	+109.4%	+167.4%	+7.45
F: Diabetic + *A. herba-alba*	+37.3%	+26.2%	+26.0%	+25.5%	+0.97
G: Diabetic + Dapagliflozin	+72.9%	+63.9%	+64.5%	+72.1%	+2.74
H: Diabetic + Combination	−16.1%	−20.4%	−21.7%	−22.2%	−0.98

**Table 6:** Percentage change in fasting blood glucose (FBG) from baseline (day 0) at each time point, and the absolute change at day 30, for each group. Values are derived from the group means presented in Table 3. Negative values indicate a decrease from baseline.

Group	Day 7	Day 14	Day 21	Day 30	Δ FBG (Day 30, mg/dL)
A: Healthy control	−4.1%	−3.4%	−6.3%	−4.8%	−4.9
B: Healthy + *A. herba-alba*	+7.3%	−5.9%	−9.4%	−12.9%	−13.8
C: Healthy + Dapagliflozin	−29.4%	−17.6%	−7.2%	+1.9%	+2.1
D: Healthy + Combination	+5.4%	+0.7%	−1.1%	−3.6%	−3.9
E: Diabetic control	+32.4%	+38.5%	+69.0%	+57.5%	+130.8
F: Diabetic + *A. herba-alba*	−12.8%	+11.5%	+5.8%	+1.3%	+2.7
G: Diabetic + Dapagliflozin	−36.8%	−29.5%	−24.8%	−21.0%	−41.0
H: Diabetic + Combination	−6.9%	−21.4%	−27.7%	−34.3%	−63.8

## Data Availability

The data presented in this study are available from the corresponding author upon reasonable request.

## Supplementary Materials

The following supporting information can be downloaded at: https://www.mdpi.com/article/10.3390/pharmaceutics18070900/s1, Supplementary Material S1: Completed ARRIVE 2.0 checklist.
