# Supplementary material for: Aqueous Artemisia herba-alba Asso Preparation as a Botanical Adjunct to Dapagliflozin: Enhanced Glycemic Control in Streptozotocin-Induced Diabetic Rats
Source: Pharmaceutics. 2026 Jul 22;18(7):900. doi: 10.3390/pharmaceutics18070900 (PMC13416393; doi:10.3390/pharmaceutics18070900)
Supplement: Supplementary file 1 [file pharmaceutics-18-00900-s001.zip › pharmaceutics-4350009-supplementary.pdf]

Supplementary Material S1: The ARRIVE Guidelines 2.0: Author Checklist

Completed for: Aqueous Artemisia preparation combined with dapagliflozin produces greater glycated hemoglobin reduction than either treatment alone in streptozotocin-induced diabetic rats

Item	Recommendation	Section / Line Number or Reason for Not Reporting
**THE ARRIVE ESSENTIAL 10**		
**1. Study design**	a. Groups being compared, including control groups. b. The experimental unit.	Materials and methods – Study design and group allocation: Eight groups (A–H; 4 healthy, 4 diabetic) described with vehicle control, Artemisia only, dapagliflozin only, and combination. Experimental unit = individual rat.
**2. Sample size**	a. Exact number of experimental units per group and total animals. b. How sample size was decided.	Materials and methods – Study design: n = 10 per group, total N = 80 rats. Abstract: Materials and methods section. Sample size was based on previous studies using the STZ model (Dayyih et al. 2021; Akbarzadeh et al. 2007). No formal a priori power calculation was performed (acknowledged in Limitations).
**3. Inclusion and exclusion criteria**	a. Criteria for including/excluding animals. b. Animals not included and why. c. Exact value of n in each group.	Materials and methods – Diabetes induction: Animals with fasting blood glucose ≥126 mg/dL (7.0 mmol/L) 72 h post-STZ injection were retained. No animals were excluded. n = 10 in all groups as stated in Tables 1 and 2 and figure legends.
**4. Randomisation**	a. Whether randomisation was used. b. Strategy to minimise confounders.	Limitations section: “The protocol of the original study did not specify randomization or blinding.” This is explicitly stated as a limitation.
**5. Blinding**	Who was aware of group allocation at each stage.	Limitations section: Blinding was not specified in the original protocol. This is explicitly acknowledged as a study limitation.
**6. Outcome measures**	a. All outcome measures defined. b. Primary outcome measure specified.	Materials and methods – Blood sampling and HbA1c measurement: Primary outcome = glycated hemoglobin (HbA1c, %) measured by immunoturbidimetric/nephelometric method using PA120 Analyzer. Secondary outcome = fasting blood glucose (glucometer, JOYCOO®).
**7. Statistical methods**	a. Statistical methods and software. b. Assessment of assumptions.	Materials and methods – Statistical analysis: Independent-samples t-tests, two-way factorial repeated-measures ANOVA (time × diabetic status), maximum likelihood with gamma regression. Normality assessed by Shapiro–Wilk test and histogram. SPSS version 21 (IBM Corp.). $\alpha = 0.05$.
**8. Experimental animals**	a. Species, strain, sex, age, weight. b. Provenance, health status.	Materials and methods – Animals and ethics statement: Male Wistar albino rats, 7–8 weeks old, mean weight 225 ± 25 g. Purchased from the animal breeding facility of the University of Jordan (Amman,

		Jordan). No genetic modifications. Healthy, naïve animals with no prior procedures.
**9. Experimental procedures**	a. What, how, what was used. b. When and how often. c. Where. d. Why (rationale).	Materials and methods – Plant material, Phytochemical characterization, Drugs and reagents, Diabetes induction, Blood sampling sections: All procedures described in detail including STZ dose (35 mg/kg IP), Artemisia dose (0.39 g/kg BID oral gavage × 30 days), dapagliflozin dose (0.143 mg/kg/day oral gavage), blood collection schedule (days 0, 7, 14, 21, 30), housing at Applied Science University facility, and rationale for each procedure.
**10. Results**	a. Summary statistics with variability. b. Effect size with CI.	Results section: All data presented as mean ± SD. Tables 1 and 2 provide complete summary statistics for all 8 groups at all 5 time points. p-values from t-tests reported. Repeated-measures ANOVA reported with exponentiated beta coefficients and 95% CI. Effect sizes (mean differences between groups) reported.
**THE RECOMMENDED SET**		
**11. Abstract**	Accurate summary: objectives, species, strain, sex, key methods, findings, conclusions.	Abstract: Structured abstract with Context, Objective, Materials & methods, Results, Discussion and Conclusion headings. Species (Wistar albino rats), sex (male), key methods (STZ induction, 30-day oral treatment, HbA1c by nephelometry), principal findings (−0.98% HbA1c with combination, $p < 0.001$), and conclusions reported.
**12. Background**	a. Scientific background and rationale. b. How species/model addresses objectives.	Introduction: Comprehensive background on T2DM, HbA1c, SGLT2 inhibitors, Artemisia ethnopharmacology, and knowledge gap (no prior combination studies). Relevance of STZ rat model discussed in Discussion – Translational considerations.
**13. Objectives**	Research question and hypotheses.	Introduction (final paragraph): "The present preclinical study aimed to compare the effects of dapagliflozin monotherapy, an aqueous Artemisia preparation monotherapy, and their combination on HbA1c in STZ-induced diabetic rats."
**14. Ethical statement**	Name of ethics committee, licence/protocol numbers.	Materials and methods – Animals and ethics statement: Institutional Animal Ethics Committee (IAEC) of Mutah University. Approval number: EC/2023-02/FP. Date of approval: 5 February 2023. FELASA guidelines adopted.
**15. Housing and husbandry**	Details of housing and husbandry conditions.	Materials and methods – Animals and ethics statement: Mutah University, temperature ~20 °C, 12-h light/12-h dark photoperiod, ad libitum standard pellet diet and tap water. No specific environmental enrichment described.
**16. Animal care and monitoring**	a. Steps to reduce pain/suffering. b. Adverse events. c. Humane endpoints.	Materials and methods – Animals and ethics statement: Blood sampling under diethyl ether inhalation anesthesia.

		Euthanasia by sodium pentobarbital overdose (150 mg/kg IP) per AVMA 2020 guidelines. No unexpected adverse events reported. Humane endpoints were not formally pre-specified; however, animals were monitored daily for signs of distress.
**17. Interpretation / scientific implications**	a. Interpret results. b. Study limitations.	Discussion: Comprehensive interpretation of combination vs. monotherapy results, comparison with published literature (Saeedan et al. 2021; Albasher et al. 2020; Moulahoum et al. 2022). Limitations section: Six specific limitations listed including lack of standardization, STZ model limitations, short study duration, no safety endpoints, no randomization/blinding, no mechanistic studies.
**18. Generalisability / translation**	Relevance to other species/human biology.	Discussion – Translational considerations: Explicitly addresses caution regarding human translation, notes STZ model does not fully recapitulate human T2DM, and discusses relevance to clinical practice and real-world prescribing patterns.
**19. Protocol registration**	Whether a protocol was prepared/registered.	No formal protocol was pre-registered prior to the study. This is acknowledged indirectly in the Limitations section ("the protocol of the original study did not specify randomization or blinding").
**20. Data access**	Statement on data availability.	Data availability statement: "The data supporting the findings of this study are available from the corresponding author upon reasonable request."
**21. Declaration of interests**	a. Conflicts of interest. b. Funding sources and funder role.	Disclosure statement: "The authors report no conflicts of interest relevant to this work." Funding: "This research was funded by Al-Zaytoonah University of Jordan, funding number 2026–2025/09/07." The funder had no role in study design, data analysis, or manuscript preparation.

Reference: *Percie du Sert N, Hurst V, Ahluwalia A, et al. The ARRIVE guidelines 2.0: Updated guidelines for reporting animal research. PLoS Biol. 2020;18(7):e3000410.*
Checklist available at: https://arriveguidelines.org/
